# Significant association of cutaneous adverse events with hydroxyurea: results from a prospective non-interventional study in *BCR-ABL1*-negative myeloproliferative neoplasms (MPN) - on behalf of the German Study Group-MPN

**DOI:** 10.1038/s41375-020-0945-3

**Published:** 2020-07-03

**Authors:** Frank Stegelmann, Kai Wille, Hannah Busen, Christiane Fuchs, Stefanie Schauer, Parvis Sadjadian, Tatjana Becker, Vera Kolatzki, Hartmut Döhner, Rudolf Stadler, German Study Group-MPN, Konstanze Döhner, Martin Griesshammer

**Affiliations:** 1 Department of Internal Medicine III, University Hospital of Ulm, Ulm, Germany; 2 University Clinic for Hematology, Oncology, Haemostaseology and Palliative Care, Johannes Wesling Medical Center Minden, University of Bochum, Minden, Germany; 3 Faculty of Business Administration and Economics, Bielefeld University, Bielefeld, Germany; 4 Institute of Computational Biology, Helmholtz Zentrum München, German Research Center for Environmental Health GmbH, Neuherberg, Germany; 5 University Clinic for Dermatology, Venereology, Allergology and Phlebology, Johannes Wesling Medical Center Minden, University of Bochum, Minden, Germany

**Keywords:** Myeloproliferative disease, Translational research, Myeloproliferative disease

## To the Editor:

Until today, hydroxyurea (HU) remains the most frequently used cytoreductive drug for long-term treatment of patients (pts) with classical *BCR-ABL1*-negative myeloproliferative neoplasms (MPN) [1]. Since HU is known to be associated with the occurrence of ulcers in a dose dependent manner, leg ulcers and mucocutaneous manifestations are defined criteria for HU intolerance by the European LeukemiaNet (ELN) [2–5]. However, the spectrum of cutaneous adverse events (CAE) caused by HU is much broader and ranges from skin dryness to malignant lesions like squamous cell carcinoma [6]. Since retrospective studies reporting HU associated CAE in 5–10% of pts may underestimate the frequency and the clinical relevance of such symptoms, we initiated a non-interventional trial focusing on prospective observation of CAE associated with different cytoreductive drugs used in routine MPN management [7–9].

We included pts with polycythemia vera (PV), essential thrombocythemia (ET), and primary or secondary myelofibrosis (MF) regularly presenting at our MPN outpatient clinics in Ulm and Minden (Germany), respectively. All pts included were diagnosed according to WHO criteria and treated with cytoreductive drugs according to ELN recommendations [10, 11]. Most frequently used agents were HU, anagrelide (ANA), conventional or pegylated interferon-alpha (IFN), and ruxolitinib (RUX). The institutional review board of each center approved the study. Prospective follow-up time was defined as the interval from giving informed consent to last visit. All data were collected in one database. Enrollment started at 09-Feb-2011 and date of last follow-up (´data cut-off´) was 09-Jan-2018. In total, 172 MPN pts have been evaluated.

Medical history was carried out by a set of specific questions at study entry and at every three-monthly visit. Possible relation between CAE and cytoreduction was assessed by the physician. One treatment course (TC) was defined as use of one cytoreductive treatment for at least one month. Combination therapy consisting of two drugs for longer than one month each was considered as two TC. The annual incidence of MPN-therapy associated CAE was calculated by dividing the number of events by the total number of patient-years. Differences in the proportions were estimated using chi square test. Skin alteration-free times between treatment groups were compared via log-rank test.

**Fig. 1:**
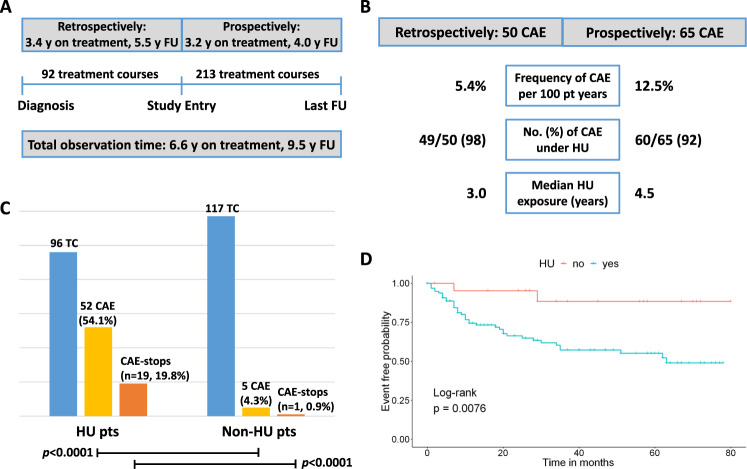
Observation times, cutaneous adverse events, and association with hydroxyurea treatment. **a** Overview of the total observation time separated into the retrospective and prospective part of the study (y years; FU follow-up time); 36 treatment courses (TC) extended from the retrospective observation period to the prospective part and were counted as prospective TC. **b** Numbers and percentages of cutaneous adverse events (CAE) in the retrospective and prospective study part (pt patient; HU hydroxyurea). **c** Number of TC, associated CAE, and treatment stops due to CAE (´CAE-stops´) in the HU and non-HU prospective cohorts. **d** Prospective CAE-free survival probability from study entry to last follow-up under HU (blue) and non-HU treatment (red).

Among 172 pts, 52% were female and median age at study entry was 62.2 years (range, 23.1–89.3). Most pts were diagnosed with PV or ET (35% each), and 25% with MF. Median retrospective observation time from MPN diagnosis to study entry was 5.5 years (range, 0.1–32.6) and median prospective follow-up time from study entry to data cut-off was 4.0 years (range, 0.1–7.2) (Fig. 1a). Furthermore, median retrospective and prospective treatment times were 3.4 years (range, 0.1–32.5) and 3.2 years (range, 0.1–5.7), respectively. In 100/172 pts (58%), treatment-free courses were observed with a median cumulative time of two years (range, 0.1–23.0).

The total number of recorded TC was 305 (retrospective, *n* = 92; prospective, *n* = 213). Of note, 36/213 TC (16.9%) extended from the retrospective observation period to the prospective part and were counted as prospective TC. The four most frequently used drugs were HU (150/305 TC, 49.2%), RUX (66/305 TC, 21.6%), ANA (50/305 TC, 16.4%), and IFN (39/305 TC, 12.8%) (Supplementary Table S1). The average number of different cytoreductive drugs per patient was 2 (range, 1–6). The vast majority of TC (300/305, 98.4%) were periods with one cytoreductive drug; combination therapies were rare (5/305, 1.6%).

Of the 115 CAE in total, 50 (43.5%) were observed retrospectively based on the medical history of the pts (Fig. 1b). The overall CAE incidence during the retrospective treatment time was 5.4% per 100 pt years. Median treatment time until occurrence of CAE was 3.4 years (range, 0.1–32.5). 49/50 CAE (98.0%) occurred under HU treatment after a median time of 3.0 years (range, 0.1–22.7). HU was discontinued in 31.5% of pts due to CAE. Only one CAE occurred under ANA after a treatment time of 0.1 years and led to discontinuation of ANA, while no CAE occurred under IFN; there were no RUX treated pts in the retrospective analysis.

During prospective observation, HU accounted for almost half of TC (96/213, 45.1%), followed by RUX (66/213, 31.0%), ANA (27/213, 12.7%), and IFN (24/213, 11.3%) (Table 1). Median treatment times since study entry were as following: HU 5.4 years (range, 0.1–21.0), RUX 1.8 years (range, 0.2–5.3), ANA 2.6 years (range, 0.1–18.0), and IFN 6.1 years (range, 0.3–22.3).

**Table 1:** Occurrence and type of 65 cutaneous adverse events (CAE) during 213 prospective treatment courses (TC) in 172 MPN patients.

	Hydroxyurea	Ruxolitinib	Anagrelide	Interferon Alpha
TC, no. (%)	96 (45.1)	66 (31.0)	27 (12.7)	24 (11.3)
Treatment time, median years (range)	5.4 (0.1–21.0)	1.8 (0.2–5.3)	2.6 (0.1–18.0)	6.1 (0.3–22.3)
TC with CAE, no. (%)	53 (55.2%)	2 (3.0%)	0 (0)	3 (12.5%)
CAE, no.	60	2	0	3
Treatment time until CAE, median years (range)	4.5 (0.2–15.3)	0.4 (0.3–0.5)	N/A	11.0 (3.8–17.9)
TC with drug discontinuation due to any reason, no. (%)	52 (54.2)	14 (21.2)	8 (29.2)	7 (29.2)
TC with drug discontinuation due to CAE, no. (%)	19 (19.8)	0 (0)	0 (0)	1 (4.2)
Type of CAE, no. (%)				
Ulcers^a^	15 (15.6)	0 (0)	0 (0)	0 (0)
Precancerous lesions	10 (10.4)	0 (0)	0 (0)	0 (0)
Skin cancer^b^	4 (4.2)	0 (0)	0 (0)	0 (0)
Various non-malignant CAE except ulcers^c^	31 (32.3)	2 (3.0)	0 (0)	3 (12.5)
Σ, no. (%)	60 (62.5)	2 (3.0)	0 (0)	3 (12.5)

^a^Leg ulcers (*n* = 12), oral ulcers (*n* = 3).

^b^Basal cell carcinoma (*n* = 3), squamous cell carcinoma (*n* = 1).

^c^HU: skin rashes (*n* = 14), skin dryness (*n* = 8), oral stomatitis (*n* = 4), nail changes (*n* = 2), erythema of the foot, psoriasis plantaris pustolosa, and livid-colored leg (*n* = 1, each).

In the prospective study part, 65/115 CAE (56.5%) were observed in 58/213 TC (27.2%) resulting in an overall incidence of CAE of 12.5% per 100 pt years (Fig. 1b). In 53/96 HU TC (55.2%) a total number of 60/65 (92.3%) drug associated CAE occurred after a median treatment time of 4.5 years (range, 0.2–15.3): ulcers (*n* = 15, 25%; leg ulcers, *n* = 12, and oral ulcers, *n* = 3), skin rashes (*n* = 14, 23%), actinic keratoses (*n* = 9, 15%), increased skin dryness (*n* = 8, 13.3%), oral stomatitis (*n* = 4, 6.7%), basal cell carcinomas (*n* = 3, 5%), pathological nail changes (*n* = 2, 3.3%), squamous cell carcinoma and dysplasia of the vulva (*n* = 1, each) (Table 1). The remaining three CAE (5%) consisted of single other dermatologic events (erythema of the foot, psoriasis pustolosa plantaris, and livid-colored leg).

At time of CAE occurrence, the median daily HU dosage was 1000 mg (range, 318–2000). The cumulative median HU dosage until appearance of first CAE was 1533 g (range, 15–7520).

During the prospective study time, HU was discontinued due to any reason in 52/96 HU TC (54.2%) and due to CAE in 19/96 HU TC (19.8%). The following CAE resulted in discontinuation in 19/52 TC (36.5%): skin ulcers (10/19 TC, 52.6%), actinic keratoses and skin rashes (3/19 TC each, 15.8%), nail changes (2/19 TC, 10.5%) and skin dryness (1/19 TC, 5.2%). Non-CAE reasons for HU discontinuation in 33/52 TC (63.5%) were disease progression (12/33, 36.4%), hematological toxicity (9/33, 27.3%), non-hematological side effects (8/33, 24.2%), and various personal reasons (4/33, 12.1%).

Regarding non-HU TC (i.e. RUX, ANA, and IFN), CAE under RUX occurred in 2/66 TC (3.0%) after a median time of 4.5 months. One pt developed a pityriasis and one pt had erythromelalgia under a daily RUX dosage of 10 mg. Neither of the two RUX associated CAE was leading to discontinuation of the drug. In contrast, no CAE occurred under ANA during the prospective study time, while CAE under IFN occurred in 3/24 (11%) TC after a median treatment time of 11 years (range, 3.8–17.9). Of these, two pts developed increased photosensitivity and one pt suffered from rash after subcutaneous application leading to IFN discontinuation.

Comparing the total numbers of prospectively recorded CAE in HU vs. non-HU TC, we found a statistically higher incidence of CAE in HU treated pts vs. non-HU pts [*p* < 0.0001; 52/96 TC (54.1%) vs. 5/117 TC (4.3%)] (Fig. 1c). With regard to treatment discontinuation, significantly more HU TC were terminated due to CAE compared to non-HU TC [*p* < 0.0001; 19/96 TC (19.8%) vs. 1/117 TC (0.9%)].

Finally, we analyzed the CAE-free survival over time during the prospective part of the study. Compared by log-rank test, a significant difference between HU and non-HU TC regarding the occurrence of CAE was observed (*p* = 0.0076; Fig. 1d). This was also true for the complete study period including the retrospective observation time (*p* = 0.0036).

In summary, the major findings of this observational study were (i) the significant association of HU with the occurrence of CAE, (ii) an overall CAE incidence twice as high when data were evaluated prospectively compared to retrospective analysis, suggesting that CAE associated with HU may be underdiagnosed and (iii) a discontinuation rate of ~20% for HU treatment due to CAE. However, it has to be taken into account that HU was the most frequent cytoreductive agent used in the pts accounting for approximately one half of all TC in both study parts.

In line with our retrospective observation part, Antonioli et al. reported a CAE rate of 4.9% under HU in a large retrospective study including more than 3400 MPN pts [9]. Here, CAE occurred after a cumulative HU dosage of about 1400 g and after a median treatment time of 60 month. Moreover, in one single-center study with prospective observation the incidence of HU associated CAE was even 60% after a median time of 3.6 years [12].

Among CAE associated with HU, ulcerous lesions (25%) and skin rashes (23%) occurred most frequently, while precancerous lesions and local skin cancers accounted for 17% and 7% of events, respectively. To prevent the occurrence of CAE under HU, we strongly recommend to (i) inform pts about possible CAE before treatment start, (ii) examine pts thoroughly before and during therapy, and (iii) advise pts about adequate skin care such as UV light protection and regular dermatologic controls.

Taken together, our non-interventional study shows a significant association between HU treatment and the development of skin toxicity, in particular with regard to ulcerous and (pre-)malignant lesions. CAE occurred after a median treatment time of 4.5 years, with a cumulative median HU dosage of 1533 g. Consequently, physicians and pts need to be informed about possible side-effects of the drug on skin and mucosa to prevent that CAE become a limiting factor for the long-term use of HU in MPN management.

## Supplementary information

Supplementary Table S1

German Study Group-MPN

## References

[1] Tefferi A, Barbui T (2012). Polycythemia vera and essential thrombocythemia: 2017 update on diagnosis, risk-stratification and management. Am J Hematol.

[2] Stahl RL, Silber R (1985). Vasculitis leg ulcers in chronic myelogenous leukemia. Am J Med.

[3] Montefusco E, Alimena G, Gastaldi R, Carlesimo OA, Valesini G, Mandelli F (1986). Unusual dermatologic toxicity of long-term therapy with hydroxyurea in chronic myelogenous leukemia. Tumori.

[4] Barosi G, Besses C, Birgegard G, Briere J, Cervantes F, Finazzi G (2007). A unified definition of clinical resistance /intolerance to hydroxycarbamide in essential thrombocythemia: results of a consensus process by an international working group. Leukemia.

[5] Barosi G, Birgegard G, Finazzi G, Griesshammer M, Harrison C, Hasselbalch H (2010). A unified definition of clinical resistance and intolerance to hydroxycarbamide in polycythemia vera and primary myelofibrosis: results of a European LeukemiaNet (ELN) consensus process. Br J Haematol.

[6] Antar A, Ishak RS, Otrock ZK, El-Majzoub N, Ghosn S, Mahfouz R (2014). Successful treatment of hydroxyurea-associated chronic leg ulcers associated with squamous cell carcinoma. Hematol Oncol Stem Cell Ther.

[7] Harrison CN, Campbell PJ, Buck G, Wheatley K, East CL, Bareford D (2005). Hydroxyurea compared with anagrelide in high-risk essential thrombocythemia. N. Engl J Med.

[8] Randi ML, Ruzzon E, Tezza F, Luzzatto G, Fabris F (2005). Toxicity and side effects of hydroxyurea used for primary thrombocythemia. Platelets.

[9] Antonioli E, Guglielmelli P, Pieri L, Finazzi M, Rumi E, Martinelli V (2012). Hydroxyurea-related toxicity in 3,411 patients with Ph-negative MPN. Am J Hematol.

[10] Vardiman JW, Thiele J, Arber DA, Brunning RD, Borowitz MJ, Porwit A (2009). The 2008 revision of the World Health Organization (WHO) classification of myeloid neoplasms and acute leukemia: rationale and important changes. Blood.

[11] Barbui T, Barosi G, Birgegard G, Cervantes F, Finazzi G, Griesshammer M (2011). Philadelphia-negative classical myeloproliferative neoplasms: critical concepts and management recommendations from European LeukemiaNet. J Clin Oncol.

[12] Beses C, Garcia-Pallarol F, Angona A, Marta B, Daniel LC, Concepción F-R (2017). Hydroxyurea mucocutaneous toxicity: a prospective cohort study of 110 ET and PV patients from a single institution. Blood.

