# Publisher Correction: Significant association of cutaneous adverse events with hydroxyurea: results from a prospective non-interventional study in *BCR-ABL1*-negative myeloproliferative neoplasms (MPN) - on behalf of the German Study Group-MPN

**DOI:** 10.1038/s41375-021-01366-3

**Published:** 2021-11-16

**Authors:** Frank Stegelmann, Kai Wille, Hannah Busen, Christiane Fuchs, Stefanie Schauer, Parvis Sadjadian, Tatjana Becker, Vera Kolatzki, Hartmut Döhner, Rudolf Stadler, German Study Group-MPN, Konstanze Döhner, Martin Griesshammer

**Affiliations:** 1 Department of Internal Medicine III, University Hospital of Ulm, Ulm, Germany; 2 University Clinic for Hematology, Oncology, Haemostaseology and Palliative Care, Johannes Wesling Medical Center Minden, University of Bochum, Minden, Germany; 3 Faculty of Business Administration and Economics, Bielefeld University, Bielefeld, Germany; 4 Institute of Computational Biology, Helmholtz Zentrum München, German Research Center for Environmental Health GmbH, Neuherberg, Germany; 5 University Clinic for Dermatology, Venereology, Allergology and Phlebology, Johannes Wesling Medical Center Minden, University of Bochum, Minden, Germany

**Keywords:** Myeloproliferative disease, Translational research, Myeloproliferative disease

Correction to: *Leukemia* 10.1038/s41375-020-0945-3, published online 3 July 2020

The article Significant association of cutaneous adverse events with hydroxyurea: results from a prospective non-interventional study in BCR-ABL1-negative myeloproliferative neoplasms (MPN) - on behalf of the German Study Group-MPN, written by Frank Stegelmann, Kai Wille, Hannah Busen, Christiane Fuchs, Stefanie Schauer, Parvis Sadjadian, Tatjana Becker, Vera Kolatzki, Hartmut Döhner, Rudolf Stadler, German Study Group-MPN, Konstanze Döhner & Martin Griesshammer, was originally published Online First without Open Access. After publication in volume 35, page 628–631 the author decided to opt for Open Choice and to make the article an Open Access publication. Therefore, the copyright of the article has been changed to © The Author(s) 2020 and the article is forthwith distributed under the terms of the Creative Commons Attribution.

