# Supplementary material for: Significant association of cutaneous adverse events with hydroxyurea: results from a prospective non-interventional study in BCR-ABL1-negative myeloproliferative neoplasms (MPN) - on behalf of the German Study Group-MPN
Source: Leukemia. 2020 Jul 3;35(2):628–31. doi: 10.1038/s41375-020-0945-3 (PMC8318877; doi:10.1038/s41375-020-0945-3)
Supplement: Supplementary file 1 — Supplementary Table S1 [file 41375_2020_945_MOESM1_ESM.docx]

**Supplementary Table S1:** Overview of 305 treatment courses (TC; retrospective and prospective) in 172 MPN patients usesd in different treatment lines**.** One TC was defined as the use of a cytoreductive drug in a single patient for at least one month. The four drugs used were hydroxyurea (HU), ruxolitinib (RUX), anagrelide (ANA), and interferon-alpha (IFN).

| **Drug** | **1st line (TC)** | **2nd line (TC)** | **3rd line (TC)** | **>3rd line (TC)** | **Sum (TC)** |
| --- | --- | --- | --- | --- | --- |
| HU | 124 | 24 | 2 | 0 | **150** |
| RUX | 9 | 37 | 17 | 3 | **66** |
| ANA | 9 | 35 | 6 | 0 | **50** |
| IFN | 24 | 12 | 2 | 1 | **39** |
| **Sum (TC)** | **166** | **108** | **27** | **4** | **305** |
