# Supplementary material for: Significant association of cutaneous adverse events with hydroxyurea: results from a prospective non-interventional study in BCR-ABL1-negative myeloproliferative neoplasms (MPN) - on behalf of the German Study Group-MPN
Source: Leukemia. 2020 Jul 3;35(2):628–31. doi: 10.1038/s41375-020-0945-3 (PMC8318877; doi:10.1038/s41375-020-0945-3)
Supplement: Supplementary file 2 — German Study Group-MPN [file 41375_2020_945_MOESM2_ESM.docx]

**Board Members of the German Study Group-MPN (GSG-MPN)**

Martin Griesshammer^1^ (Chair), Tim Brümmendorf^2^ (Chair), Konstanze Döhner^3^ (Speaker), Steffen Koschmieder^2^ (Speaker), Florian Heidel^4^ (Speaker), Susanne Isfort^2^ (Secretary), and Frank Stegelmann^3^ (Secretary)

^1^University Clinic for Hematology, Oncology, Haemostaseology and Palliative Care, Johannes Wesling Medical Center Minden, University of Bochum, Minden, Germany

^2^Department of Hematology, Oncology, Hemostaseology and Stem Cell Transplantation, Faculty of Medicine, RWTH Aachen University, Aachen, Germany

^3^Department of Internal Medicine III, University Hospital of Ulm, Ulm, Germany

^4^Department of Internal Medicine II, University Hospital Jena, Jena, Germany
